# *SRY* mutation analysis by next generation (deep) sequencing in a cohort of chromosomal Disorders of Sex Development (DSD) patients with a mosaic karyotype

**DOI:** 10.1186/1471-2350-13-108

**Published:** 2012-11-16

**Authors:** Remko Hersmus, Hans Stoop, Erin Turbitt, J Wolter Oosterhuis, Stenvert LS Drop, Andrew H Sinclair, Stefan J White, Leendert HJ Looijenga

**Affiliations:** 1 Department of Pathology, Erasmus MC, University Medical Center Rotterdam, Josephine Nefkens Institute, Daniel den Hoed Cancer Center, Rotterdam, The Netherlands; 2 Centre for Reproduction and Development, Monash Institute of Medical Research, Clayton, Victoria, Australia; 3 Department of Pediatric Endocrinology, Erasmus MC - University Medical Center Rotterdam, Sophia Children’s Hospital, Rotterdam, The Netherlands; 4 Murdoch Children’s Research Institute, and Department of Pediatrics, University of Melbourne, Royal Children’s Hospital, Melbourne, Victoria, Australia

**Keywords:** Disorders of Sex Development (DSD), Chromosomal-DSD, SRY, Next generation (deep) sequencing, Mutation

## Abstract

**Background:**

The presence of the Y-chromosome or Y chromosome-derived material is seen in 4-60% of Turner syndrome patients (Chromosomal Disorders of Sex Development (DSD)). DSD patients with specific Y-chromosomal material in their karyotype, the GonadoBlastoma on the Y-chromosome (GBY) region, have an increased risk of developing type II germ cell tumors/cancer (GCC), most likely related to TSPY. The Sex determining Region on the Y gene (*SRY*) is located on the short arm of the Y-chromosome and is the crucial switch that initiates testis determination and subsequent male development. Mutations in this gene are responsible for sex reversal in approximately 10-15% of 46,XY pure gonadal dysgenesis (46,XY DSD) cases. The majority of the mutations described are located in the central HMG domain, which is involved in the binding and bending of the DNA and harbors two nuclear localization signals. *SRY* mutations have also been found in a small number of patients with a 45,X/46,XY karyotype and might play a role in the maldevelopment of the gonads.

**Methods:**

To thoroughly investigate the presence of possible *SRY* gene mutations in mosaic DSD patients, we performed next generation (deep) sequencing on the genomic DNA of fourteen independent patients (twelve 45,X/46,XY, one 45,X/46,XX/46,XY, and one 46,XX/46,XY).

**Results and conclusions:**

The results demonstrate that aberrations in *SRY* are rare in mosaic DSD patients and therefore do not play a significant role in the etiology of the disease.

## Background

The development of a mammalian embryo into either female or male is primarily dependent on the sex chromosomal constitution, being XX and XY respectively. Normal male (46,XY) sex determination relies on the presence of the Y-chromosome, specifically on expression of *SRY* at the appropriate time and place during gonad development. Timely expression of this gene above a critical threshold is necessary to trigger testis formation [1,2]. If sufficient SRY is present, SOX9 will be up-regulated, leading to the formation of pre-Sertoli cells [3]. This will further orchestrate the formation of a functional testis, ultimately leading to the development of male primary and secondary sex characteristics [2]. In a 46,XX constitution, (i.e. the absence of the Y-chromosome and *SRY*) supportive cells in the gonad will, under the influence of FOXL2, WNT4, and RSPO1 amongst others, develop as granulosa and theca cells, leading to the formation of a functional ovary and female primary and secondary sex characteristics [2].

Turner syndrome (TS) is characterized by gonadal dysgenesis, short stature, and dysmorphic features (neck webbing amongst others). In 6 to 11% of cases a cell line with a normal or abnormal Y-chromosome is identified by standard cytogenetic techniques [4]. Patients with chromosomal DSD as a result of a 45,X/46,XY karyotype (mixed gonadal dysgenesis) may present with a wide spectrum of phenotypes ranging from normal male through ambiguous genitalia to female with a TS phenotype [5]. They are characterized by the presence of dysgenetic testis and/or streak gonads, with persistence of the Müllerian ducts and inadequate virilization, and classically have a 45,X/46,XY karyotype. Y-chromosome mosaicism may lead to virilization and modifications in the female phenotype of TS patients, although a direct correlation between presence of the Y-chromosome and gonadal differentiation pattern has not been found [6,7]. The presence of a specific region of the Y-chromosome in TS patients is correlated with an increased risk of developing a GCC, namely the GonadoBlastoma on Y region (GBY, i.e. TSPY) [8,9].

Mutations in the *SRY* gene are known to be involved in 46,XY sex reversal and are found in approximately 15% of 46,XY gonadal dysgenesis cases [10]. Most of the mutations detected are located in the HMG domain, responsible for the binding and bending of DNA, but several mutations outside of this domain have been reported. Several reports have also described mutations in the *SRY* gene in individuals with a 45,X/46,XY karyotype [11-14], suggesting an additional effect of mutant SRY in the gonadal development of these patients.

Until recently the detection of genetic variants present in <50% of cells was technically challenging, as conventional Sanger sequencing does not routinely reveal such changes. The development of next-generation sequencing technology has greatly simplified this type of analysis, as the potential to generate millions of sequence reads allows the detection and precise quantitation of low frequency variants. This approach has been used for identifying mosaic changes in a range of different samples types [15-17]. Here we describe the analysis of the *SRY* gene using the 454 GS/FLX sequencer in fourteen mosaic patients, including twelve patients with 45,X/46,XY, one patient with a 45,X/46,XX/46,XY, and one patient with a 46,XY/46,XX karyotype, to evaluate the potential role of SRY mutations in these patients.

## Results

In total fourteen chromosomal DSD patients with a mosaic karyotype were included in the study: twelve patients with a 45,X/46,XY, one patient with a 45,X/46,XX/46,XY, and one patient with a 46,XY/46,XX sex chromosomal DSD (Table 1). Age at biopsy or gonadectomy ranged from 6 months to 17 years of age (median age 3 years, Table 1). From seven patients the karyotype in peripheral blood lymphocytes was determined (cases 1–3, 5, 8, 12 and 14), and of five patients the gonadal karyotype was known (case 1–4, and 12). Conventional Sanger sequencing of SRY has been performed on genomic DNA from two patients (case 1 and 8), revealing no aberrations. Eight patients (57%) had a male, and six patients (43%) had a female gender. Histology of the gonads showed streak gonads, undifferentiated gonadal tissue, ovotesticular and testicular differentiation patterns. In one case no gonadal tissue was found (case 7), only adnexal structures (fallopian tubes, epididymis and an underdeveloped/dysplastic uterus). In one patient (case 3) a gonadoblastoma was described, being the precursor lesion of the type II germ Cell Tumor/Cancer (GCC) in the dysgenetic gonad [9]. 

**Table 1:** Overview of sex, karyotype, SRY variants and gonadal histology in mosaic DSD patients

**Case No**	**Sex**	**Karyotype (%) [%]**	**SRY Variants**	**Histology of the gonads**			**UGT**	**OT**	**NGT**	**Other**	**NA**	**Age biopsy/gonadectomy**
				**T**	**O**	**S #**						**Years**
**1**	F	45X/46XY (10%:90%) [58%/42%]				+ (L + R)						17
**2**	M	45,X/46,X,der(Y)(pter-q11.2::q11.2-pter) (44%:56%) [T:71%/29%; UGT: 63%/37%]		+ (R)			+ (L)					6 months
**3**	F	45X/46XY (56%:44%) [Y present§]					+ (GB) (L)					17
**4**	M	45X/46X iso Y (NA) [96%/4%]				+ (L)						6 months
**5**	F	45X/46XY (50%:50%) [NA]				+ (R)						16
**6**	F	45X/46X iso Y (NA) [NA]				+ (L + R)						1
**7**	M	45X/46XY (NA) [NA]							+ (R)			1
**8**	M	45X/46XY (50%:50%) [NA]		+ (R) &								9 months
**9**	M	45X/46XY (NA) [NA]									+	NA
**10**	F	45X/46XY (NA) [NA]				+ (L + R)						15
**11**	M	45X/46XY (NA) [NA]									+	3
**12**	F	46XX/46XY (94%:6%) [T:16%/84%; O: 68%/32%]						+ (NA)				1
**13**	M	45X/46XY (NA) [NA]									+	5
**14**	M	45X/46XX/46XY (39%:49%:12%) [NA]	c.49delT 21%	+ (L)								3
**Reference**	**Sex**	**Karyotype (%) [%}**	**SRY Variants**	**T**	**O**	**S**	**UGT**	**OT**	**NGT**	**Other**	**NA**	**Years**
[23]	F	45X/46XXY (65%/35%) [NA@]	p. Y3X		+ (L + R) ‡							NA
[11]	F	45X/46XY (82%/18%) [L:94%/6% R:98%/2% €]	p.S18N			+ (L + R) †						17
[11]	F	45X/46X mar (Y) (95.5%/0.5%) [100%/0% €]	p.S18N			+ (L + R) †						14
[21]	F	45X/46X psu dic (Y)(pter-q11::q11-pter) (40%/60%) [NA@]	p.R59G			+ (L + R)						24
[12]	F	45X/46XY (80%/20%) [NA]	p.N82X			+ (NA) *						24
[12]	F	45X/46XY (86%/14%) [NA]	p.L159TfsX167			+ (NA) *						20
[12]	F	45X/46XY (89%/11%) [NA]	p.Q74H			+ (L + R)						22
[14]	F	45X/46XY (15%/85%) [NA]	rs11575897							+ (GB) (L + R) $		13.5

(%): Karyotype in blood, [%]: Gonadal karyotype, GB: Gonadoblastoma, NA: Not Available, T: Testis, O: Ovary, S: Streak, Tissue, OT: OvoTestis,

Ov St: Ovarian Stroma, UGT: Undifferentiated Gonadal, NGT: No Gonadal Tissue, L: Left side, R: Right side.

# Including ovarian stroma.

§ Not further specified.

@ Blood karyotype confirmed on gonadal tissue, not further specified.

& Contains germ cells positive for OCT3/4, TSPY, SCF: at risk for malignant transformation (pre-CIS).

‡ Gonads contained primordial follicles, not further specified.

† Macroscopically streak.

€ Karyotype assessed in fibroblasts cultured from gonads.

* Stromal tissue with similarities to testicular histopathology present, not further specified.

$ Described as Left dysgenetic testis with GB, Right dysgenetic gonad.

Two different PCR products from each of the 14 different samples were generated, such that each could be identified by a specific 10 nt barcode sequence Additional file 1: Table S1). As the first 9 nt of these barcodes (plus the first 3 nt of the SRY-specific sequences) were sufficient to differentiate each of the products, we used the 10^th^ nt of the barcode to estimate the sensitivity of the assay. The advantage of using barcode sequences for this is that they were incorporated during synthesis of the primers used for generating the PCR products. This avoids any low level mosaicism that could theoretically be present in any of the samples being identified, giving misleading sensitivity estimates. Although the 10^th^ barcode nt was close to the 5’ end of the read, and potentially expected to have a low error rate, a previous study of 454 sequencing showed that there was no correlation between error rate and distance from the 5’ end for the first 100 bp of the read [17]. There were 56 different sets of reads, consisting of forward and reverse reads from two PCR products derived from 14 different samples. In total 194,680 reads contained the first 9 bp of any of the different barcodes used, of which only 143 (0.07%) contained a non-matching 10^th^ bp for the corresponding barcode (Additional file 1: Table S1).

When pooling the PCR products prior to sequencing an attempt was made to include equal amounts of each product, and analysis showed that 49/56 sets of reads were within 2x the number of reads of the corresponding mean. The sample with the lowest representation was present at only 0.9%, compared to the expected 1/14 or 7.1%. Despite this low level, an incorrect 10^th^ nt in this sample was detected in only 0.4% (2/495) of reads. These error rates are lower than previously reported figures of ~1%, presumably due to the fact that higher error rates have been shown to correlate with specific sequence features e.g. homopolymer stretches. To allow for this we set our lower threshold for variant detection at 2%.

Using this threshold, a variant in >2% of reads was identified in only one case (Sample 14, Table 1). This was a deletion of T on nucleotide position 197 in the SRY gene (c.49delT in reference sequence NM_003140.1) which was identified in 21% of sequence reads of the 45,X/46,XX/46,XY patient. Subsequent analysis of sample 14 by subcloning PCR product and analyzing 30 samples by conventional Sanger sequencing, could not confirm the deletion originally found by deep sequencing (data not shown).

## Discussion

SRY is the founding member of the SRY-like HMG box (SOX) family of transcription factors, characterized by a HMG domain [18]. It is involved in the binding and bending of DNA and contains two nuclear localization signals. Mutations in *SRY* are present in 10-15% of 46,XY DSD patients [10], and these patients have an increased risk of developing GCC, related to the presence of the GBY region (with TSPY as the most likely candidate gene), and the prolonged expression of OCT3/4 (POU5F1) in the germ cells [8,19,20]. Several authors have described mutations in *SRY* in rare cases with a mosaic sex chromosome constitution [11-13,21-23], indicating a potential involvement of SRY in abnormal gonadal development of 45,X/46,X,der(Y) patients.

However, in this study no confirmed mutations in *SRY* were identified in any of the fourteen cases analyzed. In case no. 14 with a 45,X/46,XX/46,XY karyotype, a deletion of T on position 197 of *SRY* (ref. seq. NM_003140.1) was found by deep sequencing in 21% of the sequence reads. However, subsequent analysis by sequencing subcloned PCR products only produced wild type *SRY* sequences, indicating that the original deep sequencing result was most likely a false positive. The results presented here are in agreement with, and extend the data reported by (and others summarized in) Nishi *et al*. [14], who found only one SRY polymorphism (c.561C → T) in a group of 27 patients (fourteen TS and thirteen mixed gonadal dysgenesis patients. In Table 1, next to the cases analyzed here, an overview of *SRY* mutations that have been reported in chromosomal DSD cases is shown. The results published until now, showing a *SRY* mutation in approximately 8% of cases, have all been obtained using conventional Sanger sequencing; the findings presented here show that, although analyzed with a highly sensitive sequencing technique, variations in *SRY* are not common in patients with a mosaic sex chromosomal constitution.

Shahid *et al.*[13] describes a mosaic TS patient, with gonadoblastoma, having a frameshift mutation (L94fsX180) in *SRY* which was inherited from the father. He was found to be mosaic for the *SRY* mutation and had oligoasthenozoospermia and a testicular GCC (seminoma), which are signs of mild Testicular Dysgenesis Syndrome (TDS), the underlying entity proposed by Skakkebæk *et. al.*[24]. They suggest that the presence of the mutated *SRY* gene might play a role in the development of gonadoblastoma and seminoma, being the precursor lesion and the invasive component of GCC respectively. However, in the series of samples analyzed here and published by others, no clear link between presence of *SRY* mutations and development of a gonadoblastoma in these patients can be made (Table 1 and references therein). Domenice *et al*. [22] describe a patient with partial gonadal dysgenesis and a *SRY* missense (S18N) mutation whose unaffected male relatives also harbored the mutation, showing no link between *SRY* mutation and TDS. However, a family with two sisters with 46,XY DSD, pure gonadal dysgenesis and a phenotypically normal brother has been described, in which a *SRY* frameshift mutation was found in the two sisters and in a mosaic constitution in their father. He showed signs associated with TDS; hypospadias, cryptorchidism, a testicular GCC (seminoma) and oligoasthenozoospermia, suggesting that mutations in SRY may be associated with TDS [25]. If variations in *SRY* play a significant role in TDS and the development of a testicular GCC remains unresolved, and may warrant further investigation.

It has been found in chimeric XX-XY mouse models that if the gonad contains less than 30% Y-positive cells, the gonad will develop as an ovary, suggesting a correlation between percentage of Y-containing cells and the gonadal differentiation pattern [26]. This seemed at first to be confirmed in humans [27,28], however two subsequent case reports and analysis of a larger series of samples show no correlation between the degree of gonadal mosaicism and differentiation pattern [6,29,30]. The study by Cools *et al.*[6] revealed no clear correlation between peripheral blood karyotype and gonadal karyotype, or between the gonadal karyotype and differentiation pattern found in the gonads. The inconsistency between gonadal karyotype and gonadal differentiation pattern cannot be explained by the presence of *SRY* mutations, as they are found only in rare cases, and do not seem to correlate with the differentiation pattern reported [11,12,14,21,23], even when ascertained by a highly sensitive next generation sequencing approach, as shown in this study.

## Conclusion

This is, to our knowledge, the first study using next generation sequencing to detect mutations in the *SRY* gene in chromosomal DSD patients with a mosaic karyotype. Although a highly sensitive method [15-17], no aberrations in *SRY* were detected. Including the present study, a total of 91 patients with a mosaic sex chromosomal constitution have been screened for *SRY* mutations, of which only seven (8%) showed a variation. This indicates that mutations in *SRY* are rare in chromosomal DSD patients with a mosaic karyotype and only play a role in a minority of cases.

## Methods

### Tissue and DNA samples

Anonymized tissue samples were collected from our diagnostic archives and diagnosed according to WHO standards [31] by an experienced pathologist (JWO). Use of tissue samples for scientific reasons was approved by the Medical Ethical Committee ErasmusMC (MEC 02.981 and CCR2041). Samples were used according to the “Code for Proper Secondary Use of Human Tissue in The Netherlands” as developed by the Dutch Federation of Medical Scientific Societies (FMWV (Version 2002, update 2011). Genomic DNA for sequencing was isolated from peripheral blood lymphocytes following standard protocols.

### Primer design and PCR amplification

SRY specific priming sequences were designed using reference sequence NG_011751. The complete coding sequence was covered in two overlapping PCR products, generating products of 383 bp and 372 bp. To facilitate analysis on the 454 GS/FLX sequencer (454 Life Sciences, Branford, CT, USA) the SRY-specific sequences were modified by adding a) the forward or reverse Titanium Primer and b) a 10 nucleotide multiplex identifier sequence, allowing all samples to be combined into a single reaction. All sequences are outlined in Additional file 1: Table S1. PCR amplification was carried out in 25 μl volumes, using 1.25 U Pfusion High Fidelity Enzyme per reaction. Cycle conditions were: 1 cycle of 94°C for 1 min; 35 cycles of 94°C for 30 sec, 62°C for 30 sec, 72°C for 1 min; 1 cycle of 72°C for 10 min. Samples were analyzed on a 1% agarose gel, then purified using the Agencourt AMPure XP kit (Beckman Coulter Genomics, Danvers, MA, USA) following the manufacturer’s protocol.

### Sequencing and data analysis

PCR products were pooled in equimolar concentrations and sequenced on the 454 GS/FLX sequencer (454 Life Sciences) at the Australian Genome Research Facility (Melbourne, Australia) following manufacturer’s instructions. The reads were de-multi-plexed based on the unique 10 nt MID sequence. Variant detection was performed with NextGene (SoftGenetics, State College PA, USA), using NG_011751 as the reference sequence for alignment. Only variants present in >2% of reads for a given sample were chosen for further analysis.

### PCR amplification and sequencing of sample 14

DNA was amplified using *SRY* specific primers SRY-up 5’-TTCAATTTTGTCGCAACTCTCC-3’ and SRY-rev 5’-GATCGAATGCGTTCATGGGTC-3’, generating a product of 237 bp. PCR amplification was performed using the BD Advantage 2 kit (BD Biosciences, Palo Alto, CA, USA). Cycle conditions were: 1 cycle of 95°C for 1 min; 45 cycles of 95°C for 45 sec, 57°C for 45 sec, 68°C for 1 min; 1 cycle of 68°C for 3 min. PCR product was analyzed on 1% agarose gel. Subsequently PCR product was cloned, transformed, plated and positive clones were analyzed using the TOPO TA Cloning Kit For Sequencing, following manufacturers instructions (Invitrogen, Life Technologies, Carlsbad, CA, USA). Sequences reactions were done with standard T3 and T7 primers, using the ABI PRISM BigDye Terminator Cycle Sequencing Ready Reaction kit and run on an ABI 3130xl Genetic Analyzer (Applied Biosystems, Life Techologies, Carlsbad, CA, USA) following manufacturer’s instructions. Sequences were analyzed with MutationSurveyor software (Softgenetics, State College, PA, USA) using reference sequence NG_011751.

## Competing interests

The authors declare no competing interests.

## Authors’ contributions

RH participated in study design, drafting of the manuscript, collection of samples, analyses of samples and experiments. HS participated in collection of samples, experiments and analysis. ET participated in experiments, and analysis. JWO participated in collection and analysis of samples. SD, AS, SW and LL, participated in study design, analysis and drafting of the manuscript. All authors read and approved the final manuscript.

## Authors’ information

Remko Hersmus is financially supported by Translational Research Grant Erasmus MC 2006.

## Pre-publication history

The pre-publication history for this paper can be accessed here:

http://www.biomedcentral.com/1471-2350/13/108/prepub

## Supplementary Material

Additional file 1 **Table S1.** Primers used for analyzing the 14 samples. Listed are the 56 different sequences that were used for amplifying two PCR products covering the SRY gene. Each primer consists of a 454-specific sequence, a 10 nt barcode unique for each sample (in bold), and a sequence for amplifying the SRY gene (italicised). The column “total reads” shows how many reads contained the first 9 nt of the corresponding barcode (plus the first 3 nt of the SRY primer to differentiate the two different PCR products), irrespective of the 10th nt of the barcode sequence. The column “total correct reads” shows how many reads contained the expected 10th nt of the corresponding barcode. Click here for file
